# Measles

**Published:** 1889-01-12

**Authors:** 


### MEASLES.

Dr. Arbuthnot, the celebrated physician of Queen Anne's time, in one of his numerous writings tells that before the great fire of London certain diseases were rife and mortal, "as likewise the measles." The Great Fire of London is supposed to have prevented recurrence of plague, and doubtless did much towards the sanitation of the city. But it certainly did not stop epidemics of measles, of which we have repeated experience. It is now known that measles recurs in large towns about every fourth or fifth year; the explanation given being that a fresh series of children have then arrived at the age when they are most susceptible to the epidemic. Now Dr. Arbuthnot, although living in a more pedantic age than the present one, was content to call measles, measles; a word apparently derived from the German maser a spot, a spotted appearance of the skin, being one of the principal characteristics of measles. But this did not suffice for nosologists for the term *rubeola*, a diminutive derived from *ruber*, red, was brought into use, and to this there is no valid objection, redness being also a characteristic of the measles eruption. And here, with the addition perhaps of such prefixes as mild and severe, nosologists might have ceased their labours, so far as all practical purposes are concerned. But for some reason, not very evident, measles also came to be termed *morbilli*, a diminutive derived from *morbus*, a disease; which, however, does not appear in every Latin dictionary. To the term morbilli, *mitiores* and *graviores* were also added. Then, as if this were not amply sufficient, we have applied to measles such designations as *rubeola notha*, or "spurious measles"; *rubeola sine catarrh; rubeola patrida; robella; rotheln;* "German measles;" *rubeoloid* disease; and even *roseola*. In one respect measles is not different from many other maladies—in fact, from most other maladies—inasmuch as it may assume a very mild or a very severe form, between which are numerous varieties or phases. Nosologists might have, with equal consistency, called it *morbus* as *morbilli*, the latter being a diminutive of the former word, and not applicable to a very severe type of measles. Nosologists might also have, with equal propriety, used such terms as *rubellulus*, "somewhat reddish," or *rubicundulus*, "a little reddish," as *rubella*, for they are all diminutives derived from *rubens*, red, and there are many degrees of redness in measles. Numerous names applied to one disease not only cause confusion, but sometimes lead to much unpleasantness. For example, one practitioner calls the malady measles, while another may apply the terms *rubeola* or *morbilli*. Patients not knowing that the same thing is signified, are apt to regard one or other of the practitioners as ignorant or mistaken. We recollect a life-long feud between two practitioners, because one said the disease was "cholera morbus," while the other only used the term cholera. The cholera morbus doctor won the day, and the cholera doctor was ever afterwards regarded as knowing nothing about the matter. Evidently nosologists who have tinkered at measles, entertained the erroneous idea that measles is a trivial malady; as evidenced by their fondness of diminutives in the nomenclature. Diminutives are words coined to express littleness, as *lapillus*, a little stone (Latin); *maisonette*, a little house (French); *manikin*, a little man (English); *tutella*, a little broker (Hinde). Morbilli, rubeola, &c., convey the impression of a little disease. But as regards the gravity of the malady, while the mortality is only about 2 per cent. in healthy localities, it amounts to more than 20 per cent. in unsanitary localities; and if a reference is made to the *Sunday Times* of December 16th it will be seen in an article, entitled "The Little Landlords of London," in what "dens of fever and disease" a large proportion of the poorer population of London abide. Before proceeding, it may be as well to mention that measles in animals—the pig, for instance—is quite a different malady. In helminthology the term measles is applied as a synonym of *cysticercus*, or "bladder worm," which gives diseased meat a spotted appearance, and which is the larval condition of tape-worm.

Morbilli, or measles, are very properly the only two names applied to the affection in the "Nomenclature of Disease," published by the Royal College of Physicians, and morbilli, or measles, commences as follows: There is first feverishness and cold in the head. The eyes are red, sore, watery, and the throat may feel sore. The glands under the jaw may be enlarged; there are fits of sneezing, running from the nose, cough, and, probably, pains in the limbs. The tongue is coated white with red edges. At the end of the third day, or beginning of the fourth, the rash shows first on the forehead, face, and neck, then on the trunk, and lastly on the arms and legs. The rash at first appears as small round red velvet-like spots, somewhat resembling flea bites, but not feeling raised above the surface of the skin. These extend and merge into each other, assuming semi-circular or crescentic outlines of a crimson or dark brick-red colour, and slightly raised above the surface of the skin. Three or four days after its appearance, the eruption begins to fade, and in about two days it disappears with much scurfiness of the skin. Often there is intolerable itching. If the temperature rises to 103 degrees the case is a severe one, when there is usually a peculiar characteristic odour, great debility, the tongue becoming dry and brown, and the eruption purple, is indicative of danger. In many cases there is not only cough, but bronchitis, or even inflammation of the lungs may occur. Sometimes, towards the termination, ophthalmia results, and at other times diarrhœa, or even dysentery. It is from these complications and sequelæ that the principal danger from measles arises. In many cases the cough continues after the eruption has gone, and there is remaining delicacy of the chest, from which the patient is long in recovering. "German measles," or *rotheln*, is by some regarded as a distinct disease, by others as a minor phase of ordinary measles, and much may be advanced on both sides of the question. Practically, it is of little consequence, for the treatment of measles is equally applicable to German measles, so that it signifies little if one is mistaken for the other.

The cause of measles is doubtless the communication of infective material from the sick to the healthy, either through the atmosphere—most likely in the early stages of the malady —or by the media of clothing, bedding, or other articles—most probably in the later stage of the disease. Glycerine, into which children with measles have been caused to breathe, shows numerous refractile atoms, which have also been found in certain of the tissues and in the blood after death from measles. It is not improbable these may be the *materies morbi.* Whether or not, it is certain that isolation of the sick from the healthy is the best means of staying the progress of an epidemic of measles. If measles enters a school, or a family, it is sure to spread, unless the most stringent isolation is enforced. But unfortunately, as before stated, measles is very infective during the early or catarrhal stage; before, perhaps, the nature of the malady is appreciated, or before measures of isolation can be adopted. Yet such means should never be neglected at any time. The popular idea that every child must have the measles is not correct. It is not a necessity, such as teething or growth. And although it may be well that, in popular parlance, "the measles should be got over," it is better that they should be escaped altogether. As regards treatment, during the actual attack there is little scope for medicine. Quiet rest, pure air, equable warmth, diluent drinks, and nourishment are the principal requisites. It is the absence of these essentials among the poor, which causes the mortality from measles and its sequelæ. A child with even mild measles allowed to go about is much more likely to suffer from bronchitis or pneumonia as a consequence than one kept in a properly ventilated and moderately warm room. Convalescents should have a change of room about the tenth day, and means should be taken to disinfect and purify the sick chamber.

During epidemics of measles certain precautions are advisable. Vaccination should be stopped. Schools should be closed at the earliest period. Children's parties should be postponed. The period between exposure to infection and the commencement of disease is from seven to fourteen days; and it is not safe for other children to associate with those having had measles until three weeks after the rash has disappeared, and then only if all clothing has been disinfected and washed.

